# Covariation of the endocranium and splanchnocranium during great ape ontogeny

**DOI:** 10.1371/journal.pone.0208999

**Published:** 2018-12-19

**Authors:** Nadia A. Scott, André Strauss, Jean-Jacques Hublin, Philipp Gunz, Simon Neubauer

**Affiliations:** 1 Department of Human Evolution, Max Planck Institute for Evolutionary Anthropology, Deutscher Platz, Leipzig, Germany; 2 Konrad Lorenz Institute for Evolution and Cognition Research, Martinstrasse, Klosterneuburg, Austria; Liverpool John Moores University, UNITED KINGDOM

## Abstract

That great ape endocranial shape development persists into adolescence indicates that the splanchnocranium succeeds brain growth in driving endocranial development. However, the extent of this splanchnocranial influence is unknown. We applied two-block partial least squares analyses of Procrustes shape variables on an ontogenetic series of great ape crania to explore the covariation of the endocranium (the internal braincase) and splanchnocranium (face, or viscerocranium). We hypothesized that a transition between brain growth and splanchnocranial development in the establishment of final endocranial form would be manifest as a change in the pattern of shape covariation between early and adolescent ontogeny. Our results revealed a strong pattern of covariation between endocranium and splanchnocranium, indicating that chimpanzees, gorillas, and orangutans share a common tempo and mode of morphological integration from the eruption of the deciduous dentition onwards to adulthood: a reflection of elongating endocranial shape and continuing splanchnocranial prognathism. Within this overarching pattern, we noted that species variation exists in magnitude and direction, and that the covariation between the splanchnocranium and endocranium is somewhat weaker in early infancy compared to successive age groups. When correcting our covariation analyses for allometry, we found that an ontogenetic signal remains, signifying that allometric variation alone is insufficient to account for all endocranial-splanchnocranial developmental integration. Finally, we assessed the influence of the cranial base, which acts as the interface between the face and endocranium, on the shape of the vault using thin-plate spline warping. We found that not all splanchnocranial shape changes during development are tightly integrated with endocranial shape. This suggests that while the developmental expansion of the brain is the main driver of endocranial shape during early ontogeny, endocranial development from infancy onwards is moulded by the splanchnocranium in conjunction with the neurocranium.

## Introduction

Palaeontologists have long exploited the endocranium and its negative imprint, the endocast, as a proxy with which to interrogate the size evolution of the brain in the primate fossil record [1–4]. Over the past two decades, these efforts have been greatly extended by employing virtual anthropological methods [5–20] to infer the evolution of both brain growth (an ontogenetic change in size) and development (an ontogenetic change in shape) from computed tomographic scans of extant and extinct primate endocrania in ontogenetic series [21–30]. While these described endocranial developmental changes are often taken as being wholly interpretable as brain developmental changes, several authors have stressed that the endocranium is not shaped exclusively by the brain. This is because, mechanistically, developmental and evolutionary changes in facial size and morphology, cranial bone thickness, and cranial musculature must also influence endocranial shape in addition to the tempo and mode of brain growth [24–25, 27–28, 30–34]. From this standpoint, the brain-endocranium relationship is not a true one-to-one correspondence; rather, endocranial structure is the age- and species-specific product of manifold internal (brain/meninx) and external (splanchnocranium/neurocranium/musculature) forces. Our challenge, then, is to delimit the extent of these forces in shaping the primate endocranium, a task further complicated by the likelihood that such developmental forces are liable to be bi-directional [35–38].

Development is not a stochastic process but rather a highly regulated one that produces predictable results. According to Gould [39–40] and Alberch et al. [41–42], it is far easier to regulate a resilient ontogeny than to alter it. Fundamental to this stability is conservation or canalization: an evolved robustness of developmental pathways to genetic and environmental disturbances that ultimately results in decreased adult phenotypic variance [43–46]. Yet, for novel forms to arise, these genetically optimized developmental canals cannot be wholly stable [47], but rather plastic in parts. Such plasticity has been ascribed by Gould [39] to natural selection acting upon variations in the timing and rate of development. This is of particular relevance as evidence suggests that developmental conservation (or canalization) is appreciable in the mammal cranium [48]. Within the primate order, we have shown previously that extant hominoids undergo highly conserved endocranial shape changes following the eruption of the deciduous dentition [24–25, 28], a result recently corroborated by Zollikofer and colleagues [30]. While we found that endocranial developmental patterns were overall conserved in tempo and mode, great ape species differed in the amount of expansion of the anterior and posterior cranial fossae [28]. This phenomenon is especially salient for several reasons. Firstly, such differences in the amount of shape change, together with an early (prenatal) establishment of species-specific morphology, likely drive and accentuate the disparity of adult hominoid endocranial shape. According to Keith [49], such fossal expansions are corollaries of dental eruption, masticatory musculature growth and nuchal area expansion (external factors), not brain growth (internal factor). Secondly, that these species differences emerged following the complete eruption of deciduous dentition—after the cessation of brain growth (reviewed in [26])—indicates that endocranial shape continues to change after the cessation of brain growth. These results then suggest that, during infancy, the splanchnocranium succeeds the brain in the determination of final (adult) endocranial form [50–52].

Thus, understanding the process of conservation itself, as driven by integration and allometry [48], is central to disentangling the relative contributions of internal and external morphological forces on the final establishment of species-specific endocranial form. Strong integration, or covariation between developmental modules, constrains evolution, whereas weakening covariation between modules permits independent specialization in response to differing selective pressures. Integration, therefore, can permit a fine mediation between species differentiation and canalization. As splanchnocranial and endocranial covariation balances dietary preferences on the one hand and encephalization on the other, such integration must be restrictive in channelling variation, yet permissive enough to act as a source of variation [53–55]. In mammals, cranial development must accommodate an enormous range of morphological variation in the gnathic apparatus. Because of this, we would expect mammalian cranial covariation patterns to be conserved, as was demonstrated across multiple mammalian orders [56]. Following Cheverud [57–58], such conservation of integration patterns is likely testament to extensive pleiotropy in cranial development.

As pointed out by Klingenberg [59], there is a large body of literature examining the morphological integration of the primate skull, as primates were employed as research subjects for both the revival of interest in morphological integration following Olson and Miller [60] and the development of geometric morphometrics. Using traditional and geometric methods, previous authors have consistently demonstrated that the adult primate cranium is morphologically integrated, regardless of which cranial components—e.g. face, neurocranium, cranial base, occipital bun and mandible—were subjected to analysis [37, 61–72]. Furthermore, this effect has been consistently identified in studies exploring morphological integration across ontogeny [57, 70, 73–75], which found that cranial developmental patterns are similar across great apes. Yet, while the literature on primate cranial developmental integration is indeed considerable, relatively little work has been done considering the endocranium itself as a developmental module. Bastir and colleagues [76] examined univariate brain and facial size as independent variables in a partial least squares analysis with basicranial shape, demonstrating that both values are important factors in determining basicranial orientation and morphology. Recently, Zollikofer and colleagues [30] utilized extant hominid virtual endocasts as a developmental module in order to determine the effects of cranial integration on endocranial morphology across ontogeny, with the aim to test the spatial packing and facial orientation hypotheses. Their results affirmed that endocranial shape is primarily moulded by cranial integration, with a correspondingly smaller proportion of variation attributable to brain morphology itself.

While the concepts of morphological integration and allometry are closely intertwined, allometry has been at the forefront of anatomical and evolutionary study for a much longer period of time. As summarized by Klingenberg and Marugán-Lobón[77], the definition and uses of allometry have evolved from the days of Huxley [78] to the geometric morphometrics era[79]. Klingenberg [80] divides allometry into two groups: (1) the Gould–Mosimann school, which defines allometry as the covariation of shape with size, and (2) the Huxley–Jolicoeur school, which defines allometry as the covariation among morphological features that all contain size information. For the former, allometry is implemented by a multivariate regression of shape variables on size, while for the latter this implementation is performed by plotting Procrustes form space [77, 80]. According to Gould [81–82], ontogenetic allometric pathways operate as “channels of positive constraint”; allometry, then, like integration, enables phenotypic variation along this line of least resistance and constrains it by delimiting the adult morphospace to a narrow range of shape disparity [82]. As much of shape variation can be in fact allometric variation, thus much of morphological integration can be due to the effects of allometry [77]. Allometry, therefore, can be a confounding factor in shape covariation analyses if unaccounted for. Because allometry has emerged as an important factor in hominoid endocranial development [21–25, 28], it is likely that allometry plays a crucial role in integrating endocranial development. For the present study, we correct for allometry in order to focus solely on those aspects of shape covariation that are unrelated to size variation.

Here, we explore which factors determine great ape endocranial shape variation at different stages of ontogeny. More specifically, by defining the endocranium and splanchnocranium as developmental modules, we analyze their covariation shape patterns among great ape species and across postnatal ontogeny. If splanchnocranial development determines endocranial structure following the end of brain growth, then we would expect to see a change in covariation patterns around this juncture. Based on the findings described in our previous work[28] and Zollikofer and colleagues[30], we hypothesize the following: (1) a shared ontogenetic pattern of covariation describes endocranial and splanchnocranial shape change in great apes; (2) species-specific variation in the direction and magnitude of shape change contributes to adult endocranial and splanchnocranial morphologies; (3) allometry alone is insufficient to describe the observed covariation between endocranial and splanchnocranial shape change; (4) endocranial shape changes are not driven solely by the development of the splanchnocranium and its associated changes in the cranial base.

Firstly, we use partial least squares analysis to establish whether morphological covariation between the splanchnocranial module and the endocranial module varies across ontogeny and among chimpanzees, gorillas and orangutans. Secondly, we aim to determine to what degree allometry is manifest in the observed ontogenetic shape covariation pattern, as per Klingenberg [80]. We will accomplish this by comparing centroid sizes for each developmental module, plotting Procrustes form space and by re-running our partial least squares analysis accounting for allometry, i.e. by analyzing the shape residuals for each module following regression on centroid size. Lastly, we will determine whether endocranial shape is exclusively driven by developmental integration with the splanchnocranium via the cranial base. Using thin-plate spline warping, we will test this by standardizing each vault for the effect of its accompanying cranial base, the idea being that the cranial base acts as the interface between splanchnocranium and endocranium during development [32, 83–87]. Thus, any residual vault shape variation would be indicative of forces that are independent of cranial base, and therefore splanchnocranial, development.

## Material and methods

### Sample

Our cross-sectional sample comprised computed tomographic scans of museum-housed dried crania from *Gorilla gorilla* (n = 75), *Pan troglodytes* (n = 69, including scans of two whole frozen neonates) and *Pongo pygmaeus* (n = 77), covering the lifespan from infancy to adulthood (Table 1). No permits were required for the described study, which complied with all relevant regulations. Sex attribution was drawn from museum records where available, while age groups were based on molar eruption (1 = no erupted deciduous dentition; 2 = incompletely erupted deciduous dentition; 3 = completely erupted deciduous dentition; 4 = ≥ one erupted M1; 5 = ≥ one erupted M2; 6 = ≥ one erupted M3). For full specimen information, including provenance, please see S1 Table.

**Table 1:** List of specimens by age and sex.

Dental age groups		Individuals per species (n)		
Sex	*Gorilla*	*Pongo*	*Pan*	Total
1	0	1	4	5
Female	*0*	*0*	*0*	*0*
Unknown	*0*	*0*	*4*	*4*
Male	*0*	*1*	*0*	*1*
2	3	2	3	8
Female	*1*	*0*	*2*	*3*
Unknown	*2*	*1*	*1*	*4*
Male	*0*	*1*	*0*	*1*
3	8	10	9	27
Female	*1*	*1*	*2*	*4*
Unknown	*6*	*8*	*3*	*17*
Male	*1*	*1*	*4*	*6*
4	12	11	19	42
Female	*2*	*1*	*4*	*7*
Unknown	*7*	*8*	*9*	*24*
Male	*3*	*2*	*6*	*11*
5	14	12	12	38
Female	*4*	*3*	*4*	*11*
Unknown	*4*	*6*	*3*	*13*
Male	*6*	*3*	*5*	*14*
6	38	41	22	101
Female	*12*	*15*	*6*	*33*
Unknown	*2*	*12*	*8*	*22*
Male	*24*	*14*	*8*	*46*
Total	75	77	69	221

### Segmentation and measurement

Endocasts were produced virtually using two- and three-dimensional semi-automated segmentation [14]. To measure the endocasts, we placed endocranial surface landmarks that encompassed the geometry of the endocranial cavity as delineated by the cranial fossae (anatomical landmarks = 29, plus semilandmarks on curves, based on our previous work [24]). We then measured on these same individuals anatomical landmarks (n = 23) and semilandmarks on curves that encompassed the geometry of the splanchnocranium; such division of the ectocranium into modules is inherently arbitrary and our definition here of the splanchnocranium does not comprise the mandible. For splanchnocranial and endocranial landmark information, please see Table 2; for full landmark data, see S2 and S3 Tables. Semilandmarks on curves were resampled to equivalent point count, and endocranial surface semilandmarks were calculated following the measurement protocol developed by us previously [24], in which all semilandmarks were slid iteratively so as to minimize the thin-plate bending energy of each specimen against the sample mean shape [88–89]. Generalized Procrustes analyses [90–92], whereby coordinates of the 309 endocranial and 202 facial anatomical landmarks and slid semilandmarks are transformed into Procrustes shape variables, were performed separately for endocranial and splanchnocranial landmark blocks.

**Table 2:** List of landmarks.

I. Anatomical endocranial landmarks			
*Midsagittal*	*Paired bilateral*		
Anterior sphenoid spine	Anterior clinoid process		
Foramen caecum	Optical canal		
Endobregma	Superior orbital fissure		
Endolambda	Foramen rotundum		
Internal occipital protuberance	Foramen ovale		
Opisthion	Apex of the petrous bone		
Basion	Internal acoustic meatus		
Endosphenobasion	Maximum curvature between transverse and petrous curves		
Dorsum sellae	Foramen jugulare		
	Hypoglossal canal		
II. Endocranial landmarks on curves			
*Midsagittal*	*Paired bilateral*	*Delineation*	
Midsagittal		Shape of cerebrum and cerebellum	
Clivus		Angle of the basicranium	
	Foramen magnum	Shape of foramen magnum	
	Sphenoid	Anterior from middle cranial fossae	
	Transverse sinus	Posterior cranial fossae from the vault	
	Petrous	Middle from posterior cranial fossae	
III. Anatomical splanchnocranial landmarks			
*Midsagittal*	*Paired bilateral*		
Anterior nasal spine	Root of zygomatic process		
Rhinion	Jugale		
Prosthion	Frontomalare orbitale		
Staphylion	Mastoidale		
Orale	Temporal-sphenoid suture		
	Maxillary tuberosity		
	Lingual canine margin		
	Lateral extreme of the curve, supra-orbital anterior projection		
	Medial extreme of the curve, supra-orbital anterior projection		
IV. Splanchnocranial landmarks on curves			
*Midsagittal*	*Paired bilateral*		*Delineation*
Sub-nasal clivus			Prosthion to anterior nasal spine
Midsagittal profile			Rhinion to glabella
Midsagittal palate			Staphylion to orale
	Nasal outline		Anterior nasal spine to rhinion
	Orbital rim		Frontomalare orbitale
	Zygomatic-maxillary contour		Porion to root zygomatic process
	Alveolar outline		Maxillary tuberosity to prosthion

### Analyses

**Fig 1:**
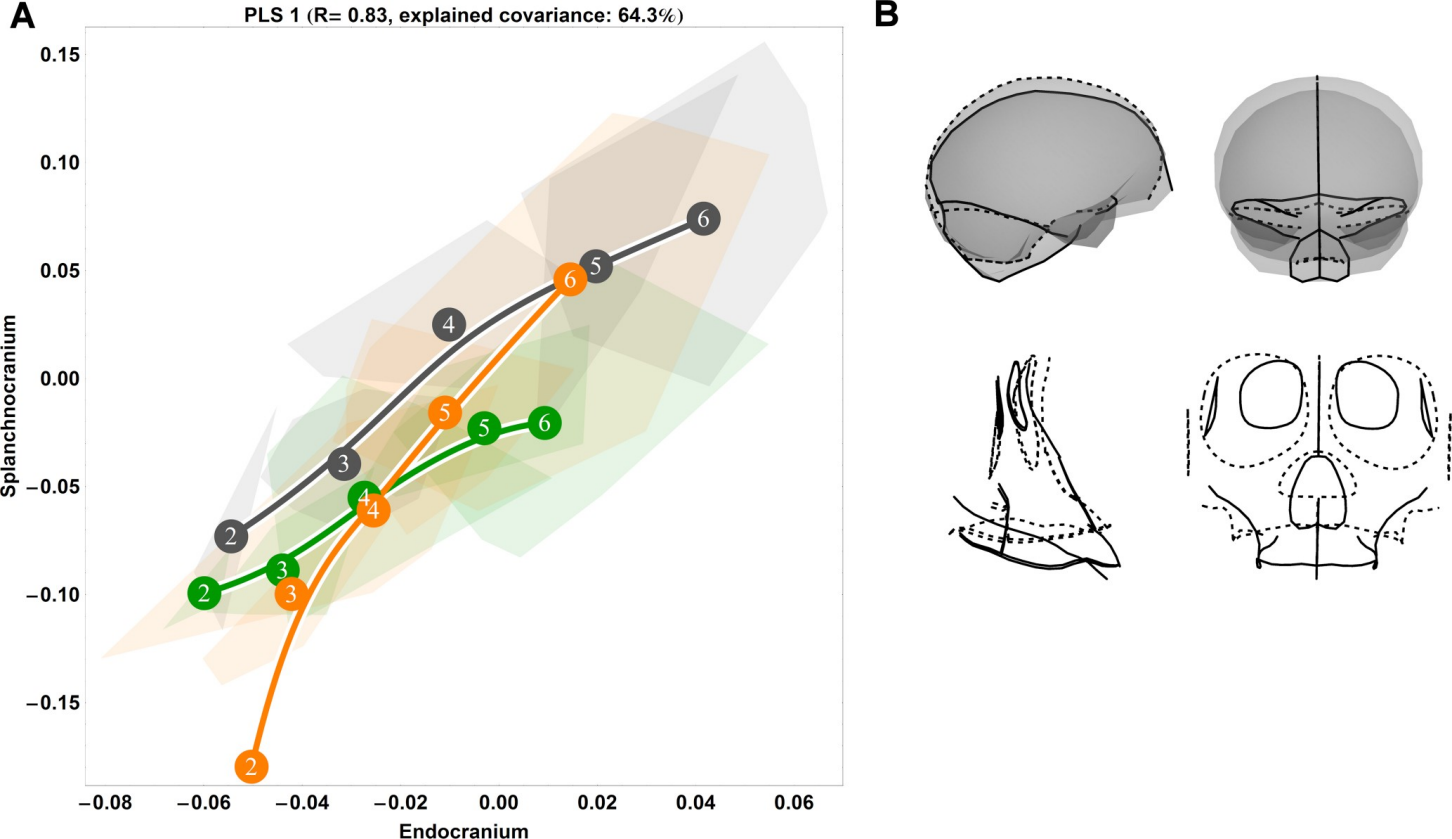
Two-block, age group mean-centred PLS analysis between endocranium and the splanchnocranium: Pooled species, singular warp 1. (A) Convex hulls represent pooled sexes of age groups 2–6 for each species; age group labels denote age group means, while lines are B-spline curves of the average species-specific trajectories. Colours: green = chimpanzee; dark grey = gorilla; orange = orangutan. (B) Sagittal (left) and coronal (right) visualizations of the shape changes corresponding to PLS 1 in the endocranium (top) and splanchnocranium (bottom), from negative (dashed line) to positive values (solid line).

To assess covariation, two-block partial least squares (PLS) analyses of Procrustes shape variables were performed. As an iterative method, this analysis yields vector pairs—singular warps, as per Rohlf and Corti [93]—ranked according to the maximum covariance between the two blocks of Procrustes shape data, here the endocranium and the splanchnocranium. In this paper, we use the terms PLS and singular warps interchangeably. Landmarks and semilandmarks were superimposed separately using Procrustes superimposition. The Procrustes shape variables were then mean-centred (standardized) by subtracting the species-specific age group mean from the shape data of each individual prior to computation of the covariance matrix, as recommended by Mitteroecker and Bookstein [94]; for species-specific analyses, this covariance matrix was composed solely of the species under investigation. A PLS analysis was performed firstly with all species combined (Fig 1 and S1 Fig). Following Bookstein and colleagues [74], individual PLS axes are termed singular warps. In this paper, we use both terms interchangeably.

**Fig 2:**
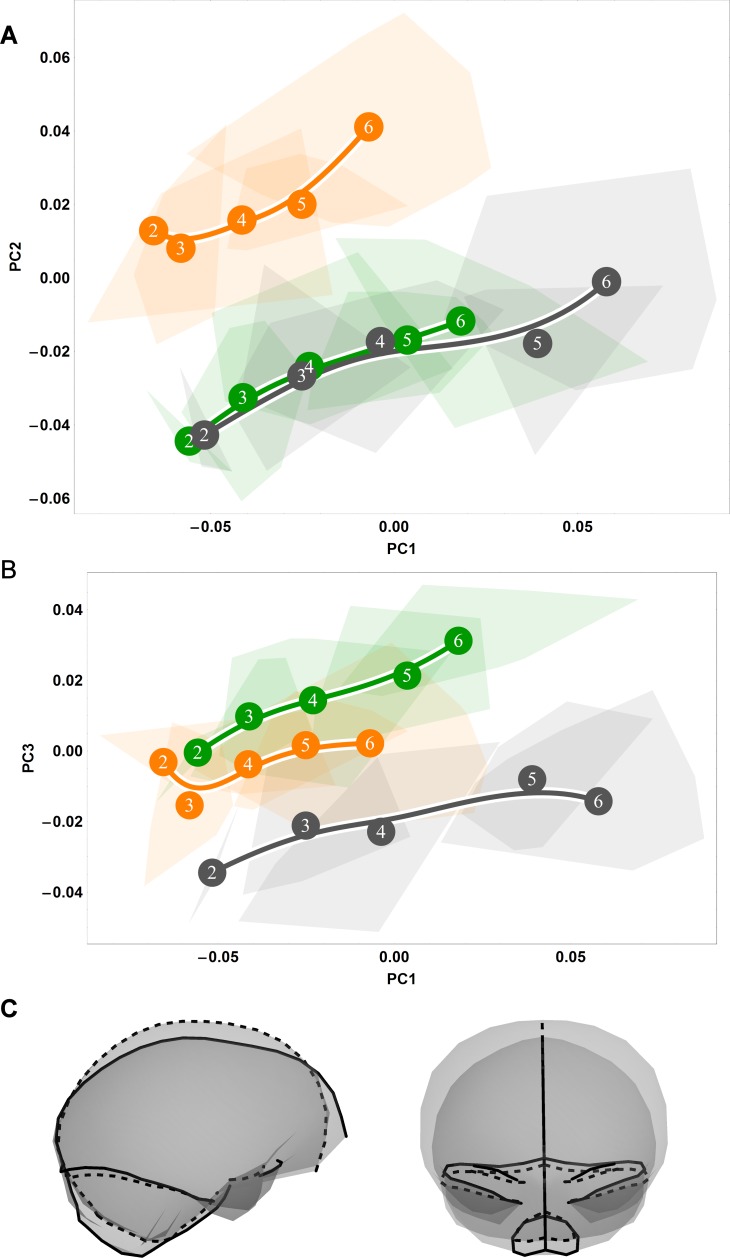
Ontogenetic shape trajectories as ontogenetic sequences of specimens in shape space: Endocranial. (A-B) Principal components analysis of Procrustes shape variables from the eruption of deciduous dentition (age group 2) to adulthood (age group 6). (A) Principal component 1 vs. principal component 2. (B) Principal component 1 vs. principal component 3. Convex hulls represent pooled sexes of age groups 2–6 for each species; age group labels denote age group means, while lines are B-spline curves of the average species-specific trajectories. Colours: green = chimpanzee; dark grey = gorilla; orange = orangutan. (C) Visualizations of sagittal (left) and coronal (right) shape changes corresponding to principal component 1, from negative values (dashed line) to positive values (solid line).

**Fig 3:**
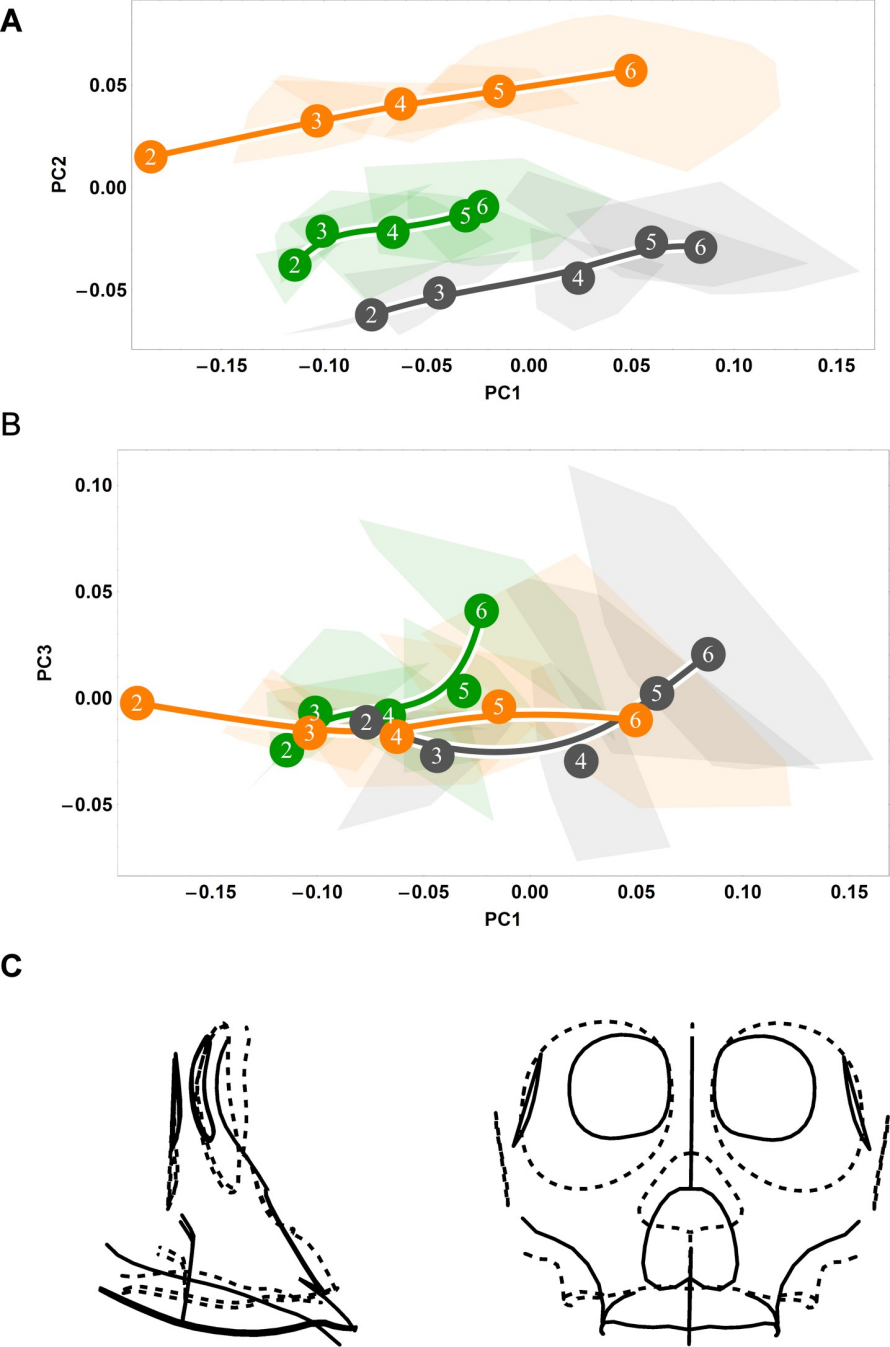
Ontogenetic shape trajectories as ontogenetic sequences of specimens in shape space: Splanchnocranial. (A-B) Principal components analysis of Procrustes shape variables from the eruption of deciduous dentition (age group 2) to adulthood (age group 6). (A) Principal component 1 vs. principal component 2. (B) Principal component 1 vs. principal component 3. Convex hulls represent pooled sexes of age groups 2–6 for each species; age group labels denote age group means, while lines are B-spline curves of the average species-specific trajectories. Colours: green = chimpanzee; dark grey = gorilla; orange = orangutan. (C) Visualizations of sagittal (left) and coronal (right) shape changes corresponding to principal component 1, from negative values (dashed line) to positive values (solid line).

**Fig 4:**
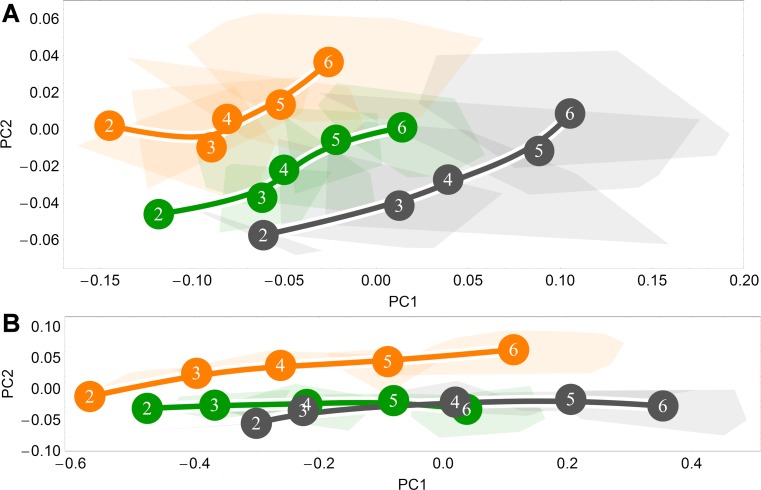
Ontogenetic shape trajectories in Procrustes form space. (A-B) Principal components analysis of Procrustes shape variables from the eruption of deciduous dentition (age group 2) to adulthood (age group 6). (A) Endocranial form space; principal component 1 vs. principal component 2. (B) Splanchnocranial form space: principal component 1 vs. principal component 2. Convex hulls represent pooled sexes of age groups 2–6 for each species; age group labels denote age group means, while lines are B-spline curves of the average species-specific trajectories. Colours: green = chimpanzee; dark grey = gorilla; orange = orangutan.

**Fig 5:**
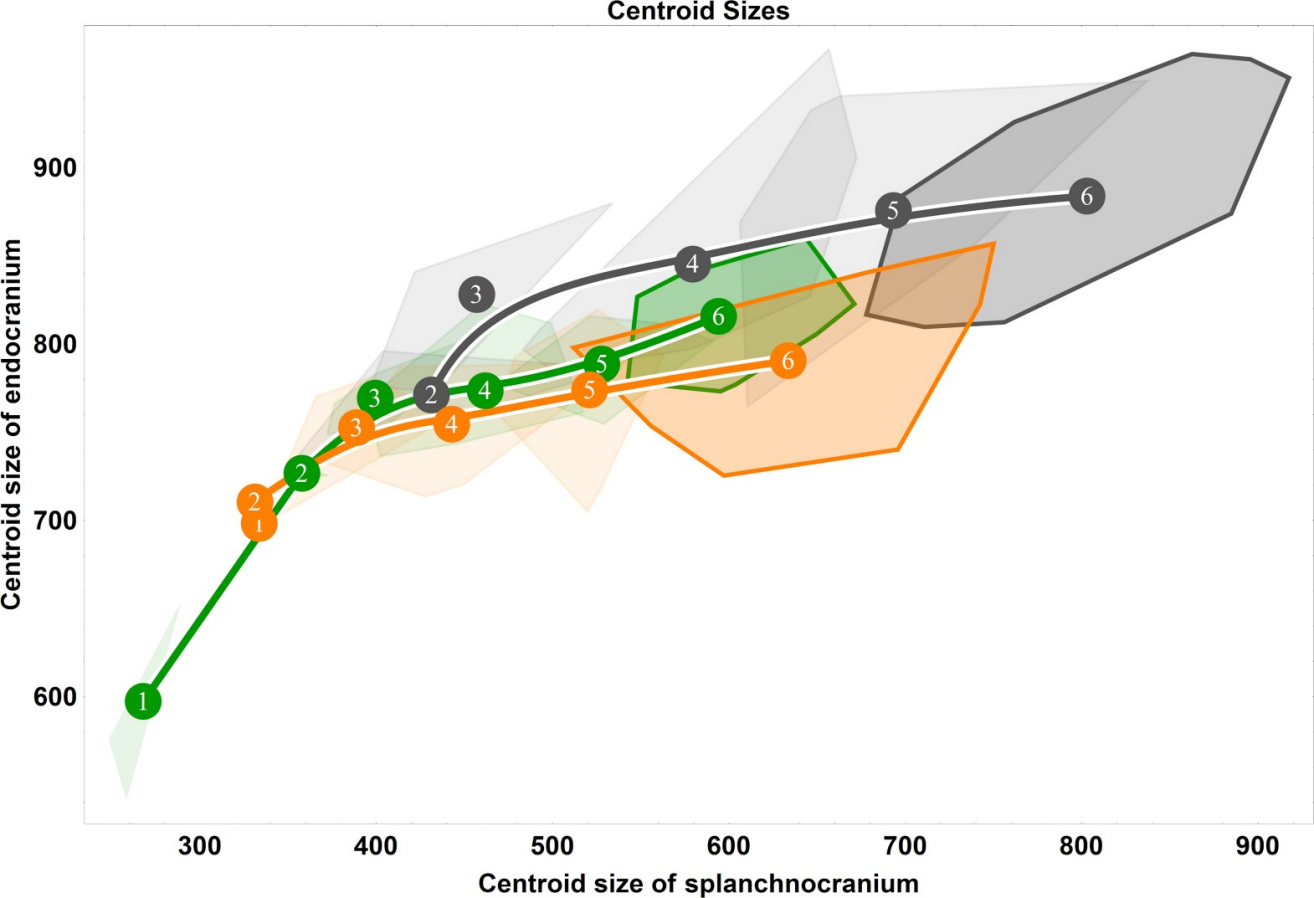
Centroid size of endocranium vs. centroid size of splanchnocranium. Convex hulls represent pooled sexes of age groups 1–6 for each species; age group labels denote age group means, while lines are B-spline curves of the average species-specific trajectories. Colours: green = chimpanzee; dark grey = gorilla; orange = orangutan.

Next, to analyze ontogenetic trajectories in shape space, Procrustes shape variables were ordinated by separate principal components analyses (PCA) for endocranial and splanchnocranial data (Figs 2 and 3). Similarly, separate principal components analyses for endocranial and splanchnocranial data were performed on Procrustes form variables to analyze ontogenetic trajectories in form space (Fig 4). Principal components (PCs) were visualized as mean shapes corresponding to the negative and positive limits of the principal components plots (Figs 2 and 3). To compare ontogenetic growth changes between modules, we plotted the centroid size of the endocranium versus the centroid size of the splanchnocranium (Fig 5).

**Fig 6:**
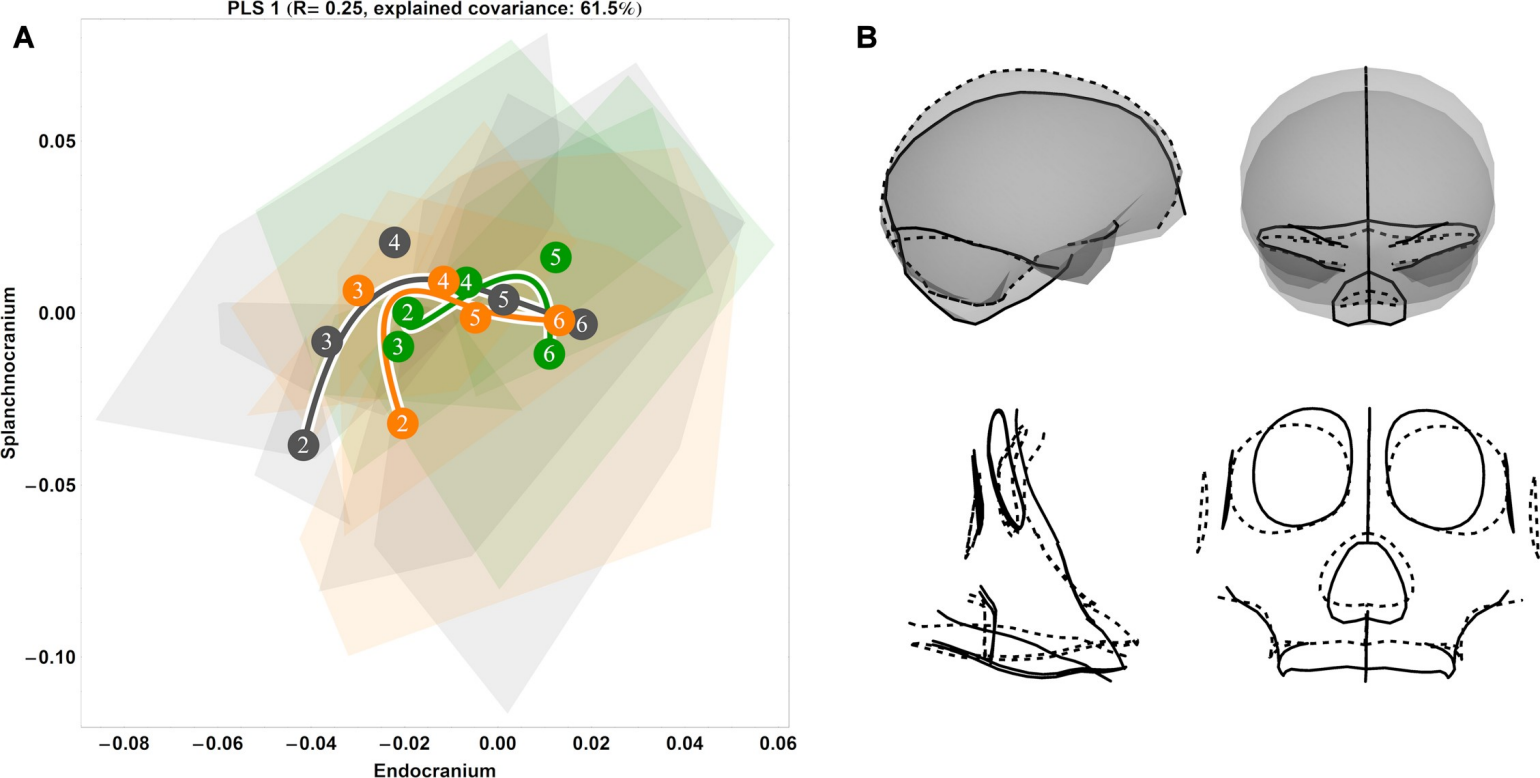
Two-block, age group mean-centred PLS analysis of endocranial and splanchnocranial shape residuals after regression on size: Pooled species, singular warp 1. (A) Convex hulls represent pooled sexes of age groups 2–6 for each species; age group labels denote age group means, while lines are B-spline curves of the average species-specific trajectories. Colours: green = chimpanzee; dark grey = gorilla; orange = orangutan. (B) Sagittal (left) and coronal (right) visualizations of the shape changes corresponding to PLS 1 in the endocranium (top) and splanchnocranium (bottom), from negative (dashed line) to positive values (solid line).

**Fig 7:**
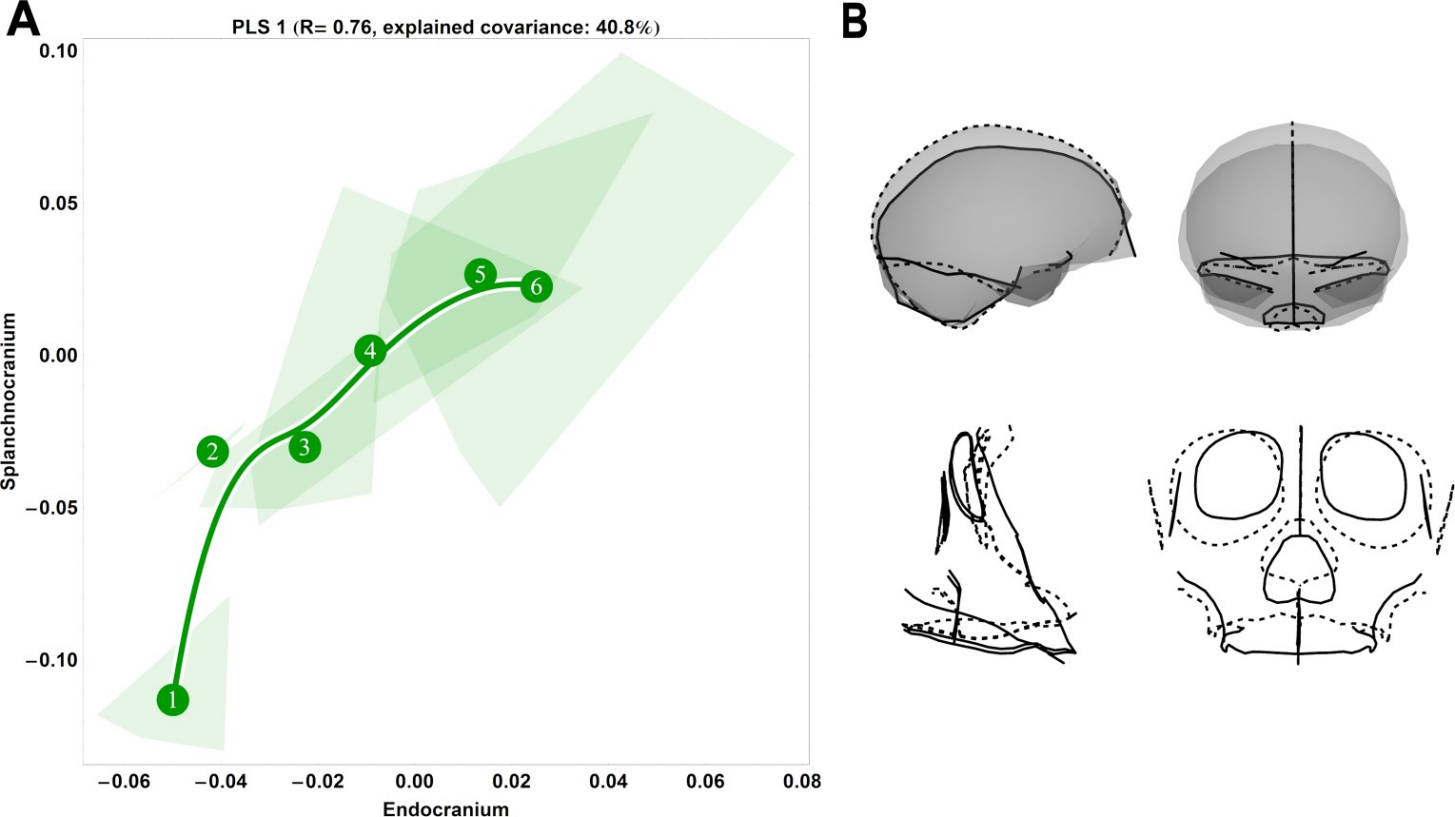
Two-block, age group mean-centred PLS analysis between endocranium and the splanchnocranium: Chimpanzees, singular warp 1. (A) Convex hulls represent pooled sexes of age groups 1–6; age group labels denote age group means, while line is B-spline curve of the average chimpanzee-specific trajectory. (B) Sagittal (left) and coronal (right) visualizations of the shape changes corresponding to PLS 1 in the endocranium (top) and splanchnocranium (bottom), from negative (dashed line) to positive values (solid line).

**Fig 8:**
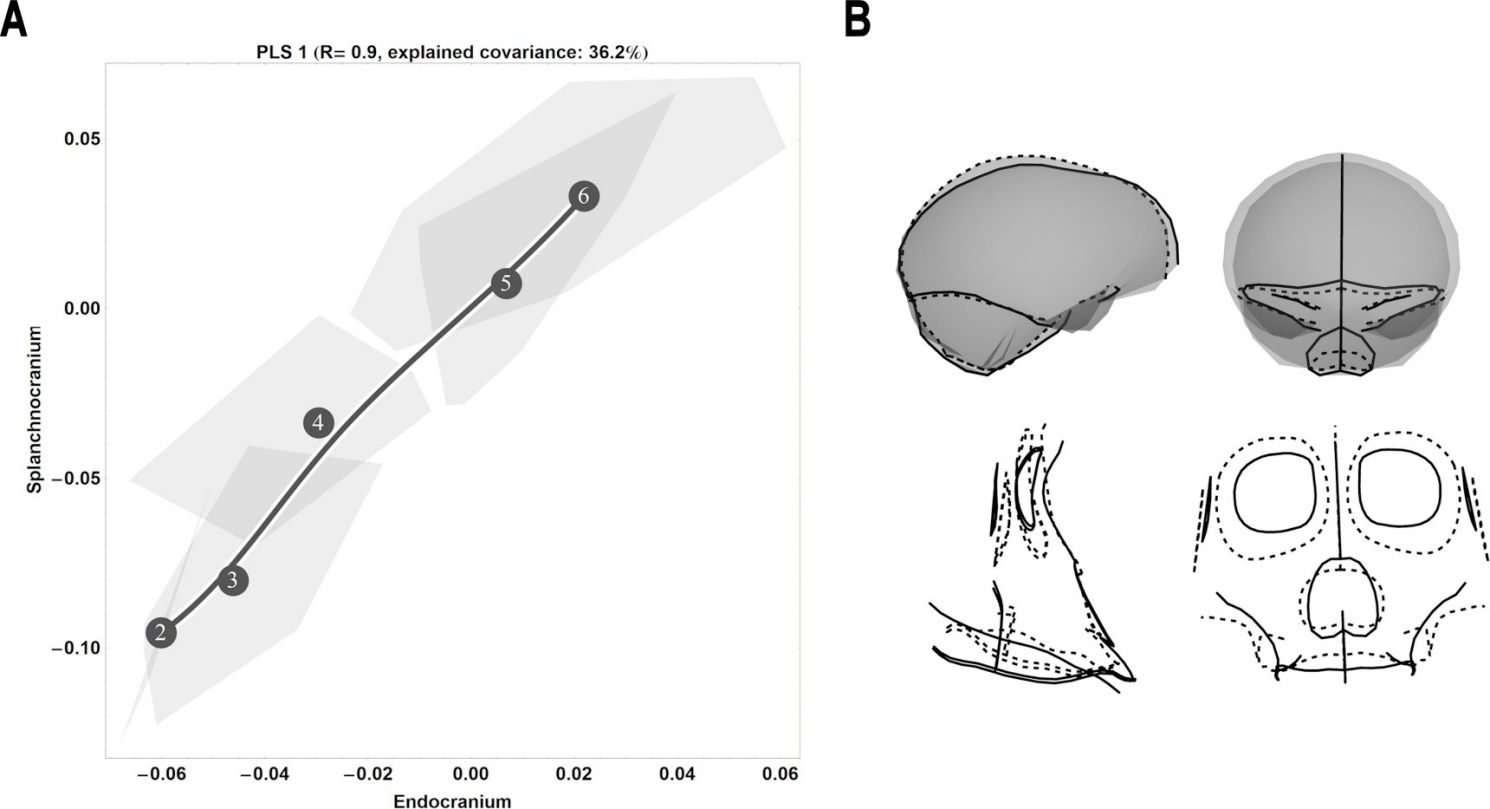
Two-block, age group mean-centred PLS analysis between endocranium and the splanchnocranium: Gorillas, singular warp 1. (A) Convex hulls represent pooled sexes of age groups 2–6; age group labels denote age group means, while line is B-spline curve of the average gorilla-specific trajectory. (B) Sagittal (left) and coronal (right) visualizations of the shape changes corresponding to PLS 1 in the endocranium (top) and splanchnocranium (bottom), from negative (dashed line) to positive values (solid line).

**Fig 9:**
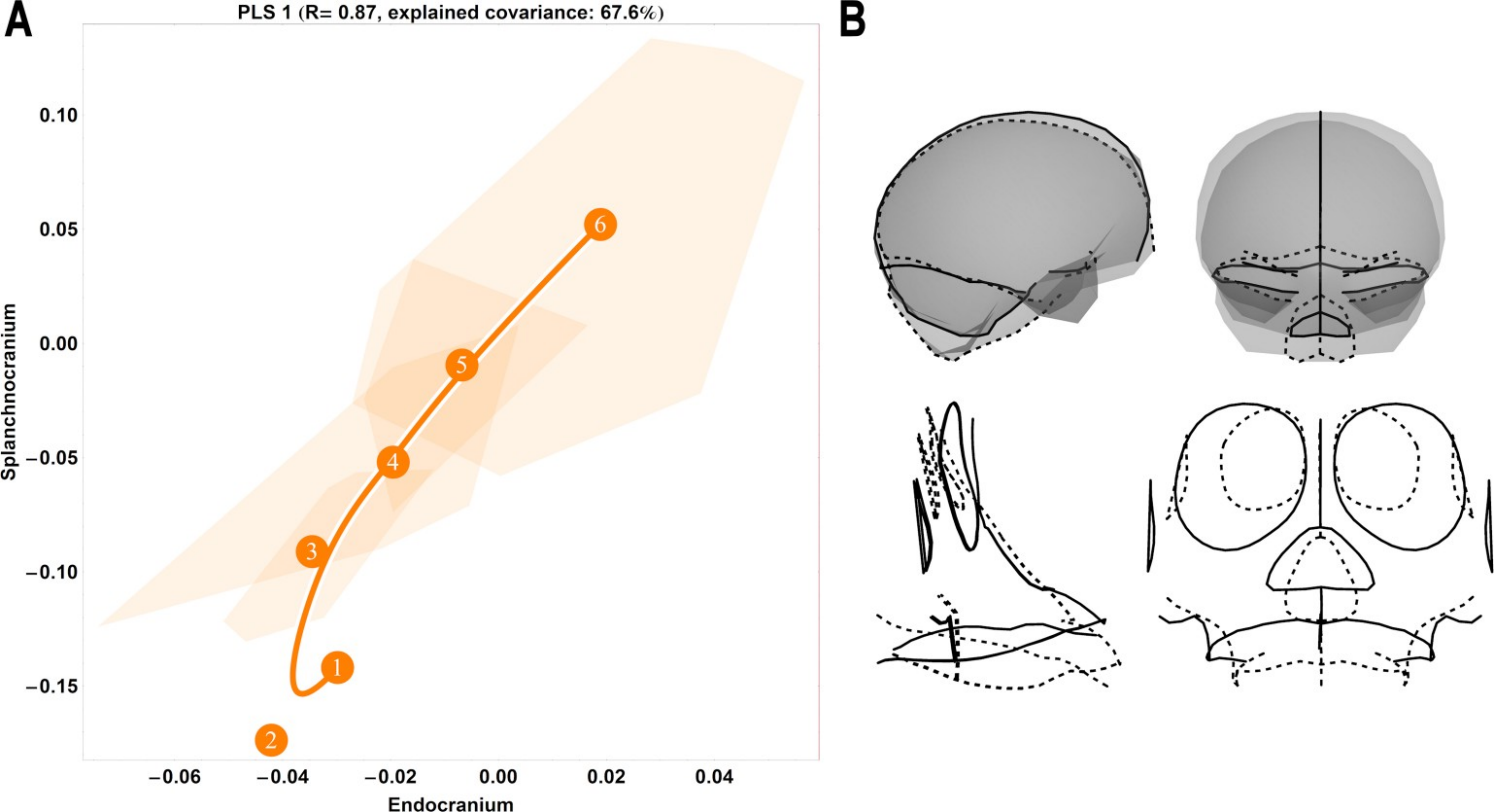
Two-block, age group mean-centred PLS analysis between endocranium and the splanchnocranium: Orangutans, singular warp 1. (A) Convex hulls represent pooled sexes of age groups 1–6; age group labels denote age group means, while line is B-spline curve of the average orangutan-specific trajectory. (B) Sagittal (left) and coronal (right) visualizations of the shape changes corresponding to PLS 1 in the endocranium (top) and splanchnocranium (bottom), from negative (dashed line) to positive values (solid line).

To determine to what degree allometry affects the PLS results, we performed an additional PLS analysis on endocranial and splanchnocranial data following regression of these data on centroid size (Fig 6 and S2 Fig). Lastly, we performed PLS analyses successively with each species separately (Figs 7–9 and S3–S5 Figs) to gain a better understanding of differences that may be obscured by pooling multiple species [94–95]. It is important to note that our assessments of trajectory similarities and differences are derived from a visual assessment, rather than additional statistical testing.

**Fig 10:**
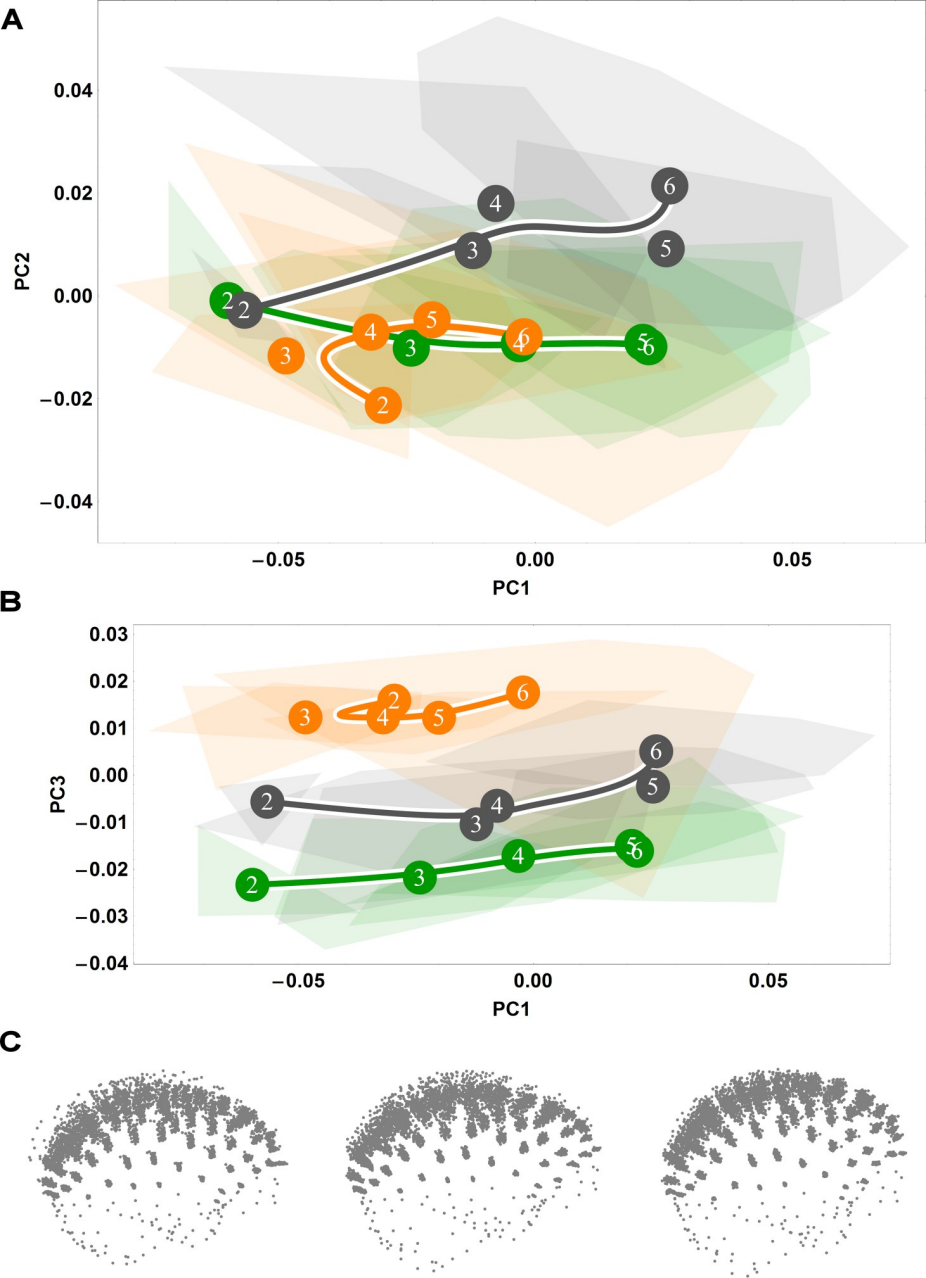
Principal components analysis of vault shape after standardizing for endocranial base shape as the interface between endocranial and splanchnocranial shapes. (A-B) Principal components analysis of Procrustes shape variables from the eruption of deciduous dentition (age group 2) to adulthood (age group 6). (A) Principal component 1 vs. principal component 2. (B) Principal component 1 vs. principal component 3. Convex hulls represent pooled sexes of age groups 2–6 for each species; age group labels denote age group means, while lines are B-spline curves of the average species-specific trajectories. Colours: green = chimpanzee; dark grey = gorilla; orange = orangutan. (C) A visualization of standardized chimpanzee (left), gorilla (middle) and orangutan (right) standardized vault shapes for each individual.

Because the braincase is tightly integrated with the face throughout development, any size and shape changes in the face also affect the endocranium, especially at the cranial base: the interface between these two cranial modules. To explore to what degree endocranial shape changes during development are driven by cranial base changes, we analyzed developmental shape changes of the cranial vault after standardizing for shape differences in the cranial base. In other words, we used geometric morphometric methods to ask: if all individuals had the exact same cranial base shape, what would their vaults look like? To this end, we first performed a Procrustes superimposition and divided our endocranial landmark set into an endocranial base component and an endocranial vault component as used previously [24]. We then removed the contribution of the endocranial base to endocranial shape by thin-plate spline (TPS) warping [96] the endocranial base shape coordinates of each specimen to the consensus shape of the endocranial base; the respective landmarks and semilandmarks on the endocranial vault were warped using this TPS interpolation [88]. Principal components analysis was then used to ordinate the resultant shape changes attributable to the cranial vault independent of the cranial base (Fig 10). If shape changes in the cranial base linked to splanchnocranial development did account for all endocranial shape changes, then a principal components analysis of these TPS-warped endocranial vaults would only reveal noise and not an ontogenetic signal.

## Results

### Ontogenetic shape covariation between species

The results of our pooled PLS analysis of covariance demonstrate that a major (*r* = 0.83) axis of covariation (singular warp 1) defines splanchnocranial and endocranial morphological changes during ontogeny, explaining 64% of the total covariance (Fig 1A). Taken as a whole, these data highlight a conserved pattern of ontogenetic integration among chimpanzees (green), gorillas (dark grey) and orangutans (orange) from the eruption of the deciduous dentition (age group 2) onwards to adulthood (age group 6). This vector shows that species differ in the amount of shape change experienced along this shared axis of covariation, whereby the magnitude (vector length) of endocranial and splanchnocranial shape changes between age group means is overall highest in orangutans and lowest in chimpanzees, with gorillas intermediate to both. For chimpanzees, the greatest amount of shape change occurs between age groups 4 and 5, while in gorillas, the greatest comparable changes are between age groups 3 and 4, and again to a lesser extent between age groups 4 and 5. Along this axis of covariation (singular warp 1), orangutans are distinct in this regard, undergoing large amounts of shape change both early in ontogeny (between age groups 2 and 3) and in late adolescence (age group 5 to 6). With respect to direction, gorilla and chimpanzee covariation trajectories parallel each other until age group 3 in gorillas and age group 4 in chimpanzees, whereupon they begin to diverge slightly. Conversely, orangutans are again exceptional to the overall pattern: the orangutan trajectory is entirely non-overlapping at age group 2, whereupon it overlaps with the African apes at age group 3; at this juncture, however, its direction continues at an angle to that of chimpanzees and gorillas.

The corresponding endocranial shape changes (Fig 1B) describe a lengthening of the anterior cranial fossae, an antero-inferior expansion of the temporal poles and a downwards rotation of the foramen magnum, while the splanchnocranial shape changes denote an antero-inferior extension of the maxilla, a proportional reduction of the orbits and an inferior extension of the nasal area. Because all specimens are scaled to unit centroid size during the Procrustes superimposition, the relative size of the orbits decreases in the visualization of the shape changes associated with singular warp 1, indicating that the actual size of the face is increasing along the positive direction of the y-axis. As the face grows and becomes more prognathic during postnatal ontogeny, the endocast becomes more elongated antero-posteriorly.

Higher PLS axes recover considerably weaker patterns of covariation between the endocranium and the splanchnocranium without a clear, interpretable ontogenetic signal (S1 Fig). From singular warp 2, accounting for just 11% of the sample covariation, it is apparent that this axis is predominately driven by ontogenetic changes in gorillas and chimpanzees, capturing little ontogenetic signal in orangutans (S1 Fig; middle). Singular warp 3 (S1 Fig; right) only explains 6% of the pooled sample covariation.

### Ontogenetic shape variation between species

To better understand the covariation patterns revealed by our partial least squares analyses in Fig 1 and S1 Fig, we next ordinated our endocranial and splanchnocranial shape data in separate principal components analyses, allowing us to examine each concomitant shape variation pattern in isolation. In endocranial shape space, the first principal component bears the strongest ontogenetic signal, separating the youngest and oldest individuals (Fig 2A). Regarding the second principal component, the African apes overlap until late adolescence, at which point gorilla endocranial shape development exceeds that of chimpanzees; orangutans are largely distinct from this African ape pattern and instead exhibit a more curved trajectory (Fig 2A). Along the third component, gorillas diverge from the general pattern at late adolescence (Fig 2B). Overall, these plots of endocranial shape space indicate that great ape trajectories differ in degree but show a marked overall likeness [28].

Splanchnocranial shape development trajectories (Fig 3) closely mirror those of the endocranium (Fig 2), both with regards to trajectory direction and magnitude. However, while it is clear that great ape trajectories exhibit overall directional similarity, diversity in magnitude is apparent along the first principal component (Fig 3A). As trajectory magnitude corresponds to the amount of shape change, this indicates that chimpanzees undergo considerably less splanchnocranial shape change development than do orangutans and gorillas in a comparative developmental timeframe (from the incomplete eruption of deciduous dentition to adulthood). Along the second principal component, orangutans are wholly distinct from the African apes (Fig 3A), while the third principal component highlights a divergence in great ape trajectory direction following adolescence (Fig 3B).

### Allometric effects

From the above PLS and Procrustes shape space results, we have observed that the great ape pattern of shape covariation (Fig 1) is manifest in the underlying similarity of the endocranial and splanchnocranial trajectories in shape space (Figs 2 and 3, respectively). However, as a considerable proportion of the ontogenetic pattern of shape development is likely attributable to allometry, that is, the proportion of shape variation due to size variation, it is interesting to remove the developmental effects that size has on shape in order to focus solely on the question at hand: how shape change alone covaries between modules with ontogeny. Therefore, to determine to what degree our PLS and PCA results are explainable by allometry, we began by examining Procrustes form, or size-shape, space whereby Procrustes shape variables and their associated centroid sizes are ordinated by principal components analysis.

From Fig 4, it is evident that both endocranial (Fig 4A) and splanchnocranial (Fig 4B) primary axes are reflective of allometric shape variation. That the parallelism of the great ape trajectories in splanchnocranial form space is oriented primarily along the first principal component indicates that this axis is driven overwhelmingly by size, with higher principal components providing little explanatory value (Fig 4B). While a similar phenomenon is apparent in endocranial form space, here the effect is less marked, with the second PC also defining a proportion of the explained variance. This indicates that size alone does not account for all endocranial shape variation.

Our form analyses indicated that size increase plays a considerable role in shape development, but to dissimilar extents in endocranial versus splanchnocranial form space. To specifically compare size increases (growth) between modules, we plotted endocranial centroid size versus splanchnocranial centroid size (Fig 5). As seen in Fig 5, all great ape species follow a similar pattern of growth, defined by a dramatic endocranial size increase from age group 1 to 2 that continues more modestly from age group 2 to 3. From age group 3 onwards (the complete eruption of the deciduous dentition), endocranial size increase is less pronounced. In contrast, splanchnocranial size increases until adulthood, with relatively regular spacing between age groups, particularly after age group 3 (Fig 5). Regarding species comparisons, chimpanzee and orangutan growth patterns overlap greatly. While gorillas start at higher initial endocranial and splanchnocranial size values at age group 2, the pattern of growth itself is overall comparable to that of the smaller apes. Adolescent and adult size variation (convex hull area) in gorillas and orangutans greatly exceeds that of chimpanzees, both endocranially and splanchnocranially, likely due to prominent sexual dimorphism in these species.

Taken together, our results in Figs 4 and 5 indicate that much of endocranial and splanchnocranial shape variation is indeed allometric shape variation. Thus, to investigate great ape ontogenetic shape covariation patterns independent of the effects of size, it is imperative to correct for allometry. To determine to what degree allometry affects the PLS results, we performed an additional PLS analysis on endocranial and splanchnocranial data following regression of these data on centroid size. By analyzing the covariation of residuals, it is clear that a large amount of the major axis of covariation (*r* = 0.25; explained covariance = 61.5%) between splanchnocranial and endocranial shape is in fact driven by allometriceffects, as interpreted by the obvious reduction of an ontogenetic signal (Fig 6) compared to the uncorrected PLS results (Fig 1). Nevertheless, some ontogenetic signal remains, defining a similar pattern between orangutans and gorillas, with chimpanzees mirroring an abbreviated version of this covariation trajectory. From this general covariation pattern, only the earliest age group (here, age group 2; incomplete eruption of deciduous dentition) deviates, indicating that covariation between endocranium and face is different in early ontogeny, but relatively consistent among species following age group 3 (complete eruption of deciduous dentition). Specifically, splanchnocranial shape change in the transition from age group 2 to 3 exceeds that of the endocranial module, whereas subsequent to age group 3, endocranial shape changes occur with little accompanying splanchnocranial shape change.

On PLS 2 (*r* = 0.41; explained covariance = 6.5%), a large amount of splanchnocranial shape change occurs between age group 2 and 3 with little concomitant endocranial shape change; here, all species trajectories are parallel (S2 Fig; middle).This indicates that covariation is markedly different (weaker) in age group 2 than in subsequent age groups. PLS 3 is less informative, with little separation of age group means or species (S2 Fig; right).

### Species-specific ontogenetic covariation

Thus far, it is evident that while great ape species are conserved with respect to an overarching pattern of shape change covariation between splanchnocranial and endocranial modules, each differs somewhat from one another; as noted, these deviations pertain mainly to variations in trajectory magnitude and direction. We further conducted PLS analyses on a covariance matrix of each species separately, which enables us to examine these species-specific covariation patterns in greater detail. Considering chimpanzees, this additionally allowed us to include a sample of neonates (age group 1), the inclusion of which permits the investigation of splanchnocranial and endocranial shape change covariation directly after birth. We found that chimpanzee shape covariationin neonates is entirely distinct from successive age groups (i.e. convex hull 1 is non-overlapping with convex hull 2), with the greatest overall amount of shape change occurring from age group 1 to 2 (PLS1; Fig 7A; *r* = 0.76). This phenomenon—a marked discontinuity between neonatal and later ontogeny—is also apparent on the third singular warp (S3 Fig; right) and is indicative of weaker covariation directly after birth: shape changes to the splanchnocranium are unmatched by comparable changes to the endocranium. On singular warp 2, covariation is also weak between age groups 1 and 2, yet here the opposite effect is observable, as a large amount of splanchnocranial shape change occurs with little corresponding shape change in the endocranium. On singular warps 1 and 3, shape covariation is greatest in adults, with relatively more shape change occurring in the splanchnocranium than in the endocranium, than in preceding age groups.

Along the first singular warp, visualizations of the shape changes of the splanchnocranium describe the difference between a relatively large-orbited splanchnocranium with no prognathism to a more prognathic adult skull with vertically compressed orbits (Fig 7B). From age group 2 to adulthood, the endocranium becomes more anterio-posteriorly elongated, the middle cranial fossae expand laterally, and the temporal poles rotate medially.

In gorillas, the ontogenetic covariation pattern along the first singular warp is both strong and positive (*r* = 0.9; Fig 8A), revealing a large amount of shape changes from age group 4 to 5,with a lesser amount from age group 3 to 4. Here, the covariation pattern is similar across ontogeny, with comparable amounts of shape change experienced in both the endocranium and splanchnocranium. Higher singular warps carry a clear ontogenetic signal, underscoring in particular a large amount of splanchnocranial shape change in early ontogeny (age group 2 to 3) that occurs with relatively less concomitant endocranial shape change (S4 Fig; middle and right).Shape-wise, the positive end of the first singular warp can be visualized as a narrow, oblong endocranium with an extended clivus, a splanchnocranium with angled zygomatic arches and an anteriorly extended maxilla; at the negative end, the endocranium is superiorly and laterally expanded in the middle and anterior cranial fossae, with an attenuated clivus, while the splanchnocranium has relatively larger orbits, a reduced maxilla, and a reduced angle along the zygomatic arches (Fig 8B).

In orangutans, the covariation pattern along the first singular warp (Fig 9A; *r* = 0.87) is more curvilinear than that of the African apes; such curvilinearity is also observable on higher singular warps (S4 Fig; middle and right). Along the first singular warp, covariation is strong, such that comparable amounts of shape change are experienced in both splanchnocranial and endocranial developmental modules. The amounts of shape change experienced between age group means is remarkably comparable across the ontogenetic signal revealed by the first singular warp (Fig 9A); on higher singular warps, this spacing is more irregular, with the greatest amounts of shape change occurring in adolescent ontogeny. As with our chimpanzee analysis, conducting a PLS of orangutans alone enabled us to include one orangutan neonate in our sample. Our results indicate that neonatal shape covariation is generally comparable to that of individuals in age group 2 on higher singular warps, but plots “off” of the general trajectory; this is likely a factor of the small sample size.

When visualized, endocranial shape variation along the first singular warp from negative to positive evinces an endocranium with a short clivus and laterally expanded anterior cranial fossae to an endocranium with inferiorly expanded temporal poles and a foramen magnum with accompanying large reductions to the lateral regions of the anterior cranial fossae (Fig 9B). In the splanchnocranium, these changes covary with a transition from relatively large orbits and a short maxilla to inferiorly elongated nasal and maxillary regions.

### Standardizing the cranial base

To determine to what extent endocranial shape change occurs independently of cranial base shape change, we standardized the Procrustes shape variables of the endocranial vault to the cranial base. The resultant trajectories in shape space show a clear ontogenetic signal (i.e. they are not static) and are relatively similar between great apes (Fig 10), but to a lesser extent than in the non-standardized analysis (Fig 2). In the projection of the first two PCs, the trajectories of chimpanzees and gorillas closely overlap at age group 2, but diverge noticeably following this juncture (Fig 10A). The orangutan shape trajectory, meanwhile, is completely distinct from the African apes until age group 4, whereupon the orangutan trajectory overlaps that of chimpanzees (Fig 10A). That the orangutan age group 2 plots “off” the trajectory for age groups 3 and 4 is probably less an indicator of a biological difference than a sampling artifact. In the projection of the first and third PCs, the African ape trajectories are approximately linear (Fig 10B), converging gradually from age group 2 onwards. Orangutans, while occupying a parallel space in this projection, again exhibit a curved trajectory between age groups 2 and 4 (Fig 10B). Overall, the spacing between age group means is similar between the African ape species: a large amount of shape change between age groups two and three and remarkably little shape change between age groups five and six. In comparison, the spacing between age group means in orangutans is more evenly distributed.

## Discussion

In our analysis of endocranial and splanchnocranial morphological integration, we have shown that great ape species are typified by similar developmental covariation patterns. Hence, in corroboration of the work of Zollikofer and colleagues [30], our results signify that conservation, or canalization, is indeed a hallmark of endocranial/splanchnocranial integration. This adds to the growing body of work demonstrating (a) that the primate cranium is highly integrated [61, 63–72]; and (b) that developmental integration patterns are comparable across primate species [57, 73–74, 94].

### Species-specific variation

Within this overarching pattern of similar developmental trajectories, some species-specific variation in magnitude and direction exists. However, as our partial least squares and principal components analyses indicate that developmental trajectories are curvilinear, this precludes the calculation of angles between trajectories to determine whether apparent species variation in vector direction is statistically significant [28]. Based on visual inspection, however, it is apparent that chimpanzees and gorillas are more similar to each other with regards to trajectory direction than either is to orangutans; this phenomenon is evident both for shape covariation and shape variation. Indeed, that chimpanzee and gorillas overlap to a considerable degree highlights that they differ in vector length between age group means: the amount of shape change covariation experienced between comparable dental-age stages. Thus, while the African apes overlap in age group means at the incomplete eruption of deciduous dentition, gorillas undergo much greater amounts of shape change such that the gorilla age group 5 mean overlaps with that of the chimpanzee age group 6 mean. Our results therefore add further substantiation to evidence showing that many aspects of the overall cranial shape differences between gorillas and chimpanzees can be explained by ontogenetic allometric scaling [97–103]. Meanwhile, it is intriguing that the amount of shape change covariation is remarkably consistent across orangutan ontogeny, as this would suggest that any growth spurt is not particularly marked. A more likely explanation, however, is that the substantial amount of covariation observed in orangutans (large convex hull area) masks multiple species, sexual bimaturism [104–108], and the development of secondary sexual characteristics [109], all of which contribute to the enormous shape disparity of orangutans [110–112]. It is likely that the observed discrepancy between orangutan and African ape trajectories is attributable to the differing facial architecture of orangutans [32, 52, 113–116].

### Effects of allometric variation

Our form space analyses indicated that size increase plays a considerable role in shape development, with this effect differing in extent in endocranial versus splanchnocranial form space. Specifically, that splanchnocranial trajectories in form space are parallel to the first axis of variation indicates that shape variation in the splanchnocranium is predominated by size variation. Such a pronounced effect is not observed in endocranial form space, implying that endocranial shape variation is not entirely determined by allometric variation [29]. By regressing out size and subsequently analyzing the covariation of residuals (Fig 6), we demonstrated that a large amount of the major axis of covariation between splanchnocranial and endocranial shape is driven by allometric effects. Despite this, an ontogenetic signal remains. This residual signal evinces comparable trajectories between great apes, particularly between orangutans and gorillas, with chimpanzees again displaying an abbreviated version of the gorilla trajectory.

### Differences between early and adolescent ontogeny

Based on virtual endocasts, we [28] and Zollikofer and colleagues [30] previously showed that great ape endocranial shape continues to develop past a point when brain growth would be expected to cease. This phenomenon is manifest in earlier studies relying solely on cranial cross-sections [32, 50–52, 117–118], and is indicative of the successive influence of the face on the continuing development of the primate endocranium. In this paper, we hypothesized that this transition between brain growth and splanchnocranial growth and development would be reflected in a change in the ontogenetic shape covariation pattern between endocranial and splanchnocranial modules. From our partial least squares analyses (Fig 1 and Figs 6–9), we demonstrated that our youngest age groups (i.e. prior to the complete eruption of the deciduous dentition) deviate to some extent from the pattern adhered to by older age groups. This corroborates our hypothesis that covariation between endocranium and face is different in early ontogeny. However, sample sizes are too small to permit statistical testing of differences in angle between early and late ontogeny for each species.

### Influence of the splanchnocranium on endocranial shape development

If endocranial shape change were primarily attributable to the development of the face and its attendant effects on the cranial base, then one would expect minimal shape change in the endocranial vault past cessation of brain growth after accounting for the shape changes of the cranial base. When we standardize endocranial shape variables to the cranial base, however, all great apes display continued shape change from the eruption of the deciduous dentition to adulthood (Fig 10). These trajectories are similar in direction between great apes, indicating a conservation of shape change in these expansionary structures following the complete eruption of the deciduous dentition (Fig 10). The differences between our cranial base-standardized (Fig 10) and non-standardized analyses (Fig 2) suggest that several of the endocranial shape changes visualized here, notably, the inferior expansion of the clivus region, the posterior migration of opisthion, and the superior rotation of the cribriform plate (Fig 2B), are corollary to the development of the face. Furthermore, our cranial base-standardized results show that while gorillas and chimpanzees follow similar trajectories in terms of direction, they differ in magnitude such that gorillas change shape more during the period of development from age groups two to three alone than do chimpanzees from age groups two to six. The ontogenetic signal from age group 3 to 6 along the major axis of covariation between endocranial and facial shape therefore takes place during a time when the relationship of low endocranial size increase and high splanchnocranial size increase is quite constant.

From the eruption of the deciduous dentition to adulthood, endocranial shape becomes more elongated as the splanchnocranium grows and become more prognathic (Fig 1 and Figs 6–9). We interpret this consistent pattern of covariation as ontogenetic integration between the splanchnocranium and the endocranium. That endocranial shape development persists to adulthood in close conjunction with splanchnocranial shape development would suggest that the latter drives the former. However, not all splanchnocranial shape changes are tightly integrated with endocranial shape. Indeed, in all of our PLS analyses here, only the first singular warp can be linked to covariation between splanchnocranium and endocranium, while higher singular warps indicate that some splanchnocranial changes during ontogeny do not affect endocranial shape. In particular, during early postnatal ontogeny, from age group 1 to 2, and from age group 2 to 3, the covariation between facial growth and development and the shape of the endocranium is weak. This time-window coincides with a period of high brain growth rates, therefore indicating that, during early postnatal development, the shape of the endocranium is primarily driven by the tempo and mode of brain expansion [21, 23–25]. At later stages of development, splanchnocranial and endocranial shape development were found to covary more strongly in all examined great ape species, particularly from the complete eruption of the deciduous dentition onwards.

## Conclusion

In summary, we have shown that covariation between the hominid splanchnocranium and endocranium is weak during early infancy but strong in subsequent age groups. This suggests that whereas the developmental expansion of the brain is the main driver of endocranial shape during early ontogeny, the adult endocranium is shaped by strong integration of the neurocranium with the splanchnocranium in later developmental periods. Throughout hominoid evolution, endocranial shape has accordingly been modified to accommodate the adaptive diversification of the face, suggesting that final endocranial form is a product of multiple developmental pathways and multiple selective pressures.

As our partial least squares analyses of orangutans and chimpanzees alone indicate, neonatal shape covariation differs from that of later age groups. In chimpanzees, for which we have more than one neonatal specimen, it is apparent that neonates are entirely distinct from the general pattern of covariation. By improving the sample composition of neonatal specimens in future research, it will be possible to ascertain the degree to which neonates differ in their morphological integration. Including prenatal data would further illuminate whether our neonatal results found here represent a pattern established prior to birth.

## Supporting information

S1 Fig Two-block, age group mean-centred PLS analysis between endocranium and the splanchnocranium: Pooled species, singular warps 1–3. Convex hulls represent pooled sexes of age groups 2–6 for each species; age group labels denote age group means, while lines are B-spline curves of the average species-specific trajectories. Colours: green = chimpanzee; dark grey = gorilla; orange = orangutan.
(TIFF)

S2 Fig Two-block, age group mean-centred PLS analysis of endocranial and splanchnocranial shape residuals after regression on size: Pooled species, singular warps 1–3. Convex hulls represent pooled sexes of age groups 2–6 for each species; age group labels denote age group means, while lines are B-spline curves of the average species-specific trajectories. Colours: green = chimpanzee; dark grey = gorilla; orange = orangutan.
(TIFF)

S3 Fig Two-block, age group mean-centred PLS analysis between endocranium and the splanchnocranium: Chimpanzees, singular warps 1–3. Convex hulls represent pooled sexes of age groups 1–6; age group labels denote age group means, while line is B-spline curve of the average chimpanzee-specific trajectory.
(TIFF)

S4 Fig Two-block, age group mean-centred PLS analysis between endocranium and the splanchnocranium: Gorillas, singular warps 1–3. Convex hulls represent pooled sexes of age groups 2–6; age group labels denote age group means, while line is B-spline curve of the average gorilla-specific trajectory.
(TIFF)

S5 Fig Two-block, age group mean-centred PLS analysis between endocranium and the splanchnocranium: Orangutans, singular warps 1–3. Convex hulls represent pooled sexes of age groups 1–6; age group labels denote age group means, while line is B-spline curve of the average orangutan-specific trajectory.
(TIFF)

S1 Table Index of specimens. Includes specimen ID and provenance information.
(XLSX)

S2 Table Endocranial landmark data, index and specimen number. File is an Excel spreadsheet, specimens are ordered as in index (S1 Table).
(XLS)

S3 Table Splanchnocranial landmark data, index and specimen number. File is an Excel spreadsheet, specimens are ordered as in index (S1 Table).
(XLS)
