# Supplementary figures and images for: Covariation of the endocranium and splanchnocranium during great ape ontogeny
Source: PLoS One. 2018 Dec 19;13(12):e0208999. doi: 10.1371/journal.pone.0208999 (PMC6300334; doi:10.1371/journal.pone.0208999)

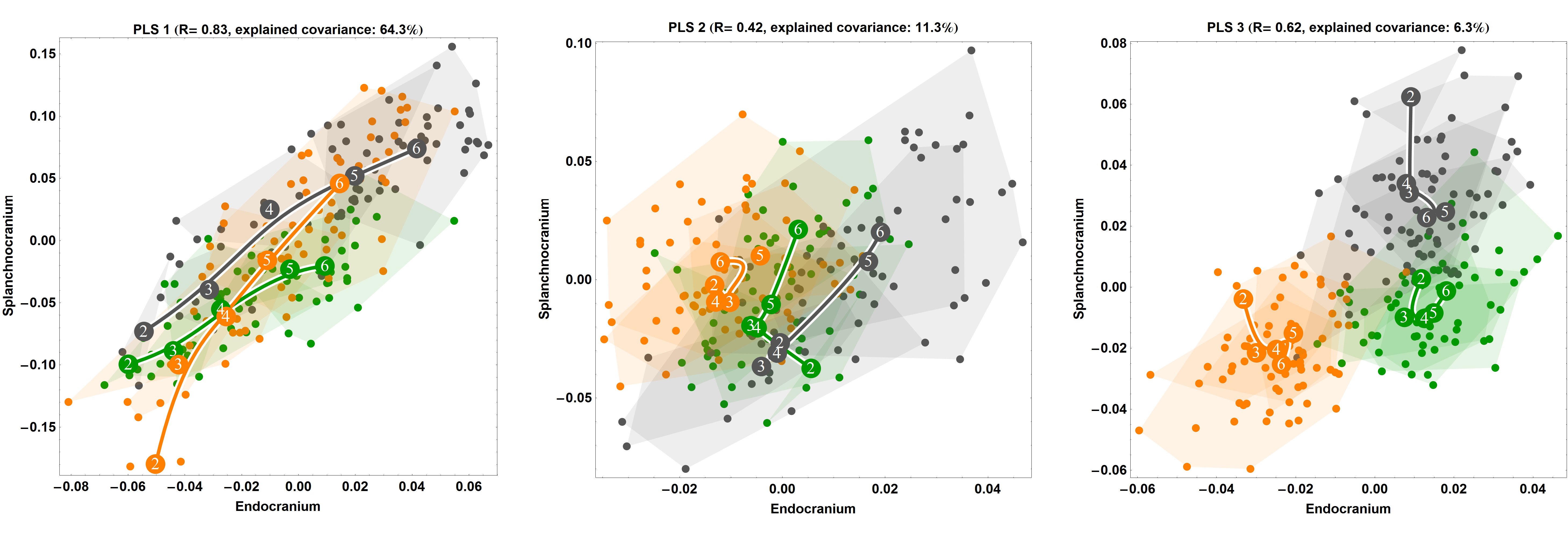

Supplement: S1 Fig — Convex hulls represent pooled sexes of age groups 2–6 for each species; age group labels denote age group means, while lines are B-spline curves of the average species-specific trajectories. Colours: green = chimpanzee; dark grey = gorilla; orange = orangutan. (TIFF) [file pone.0208999.s001.tiff]

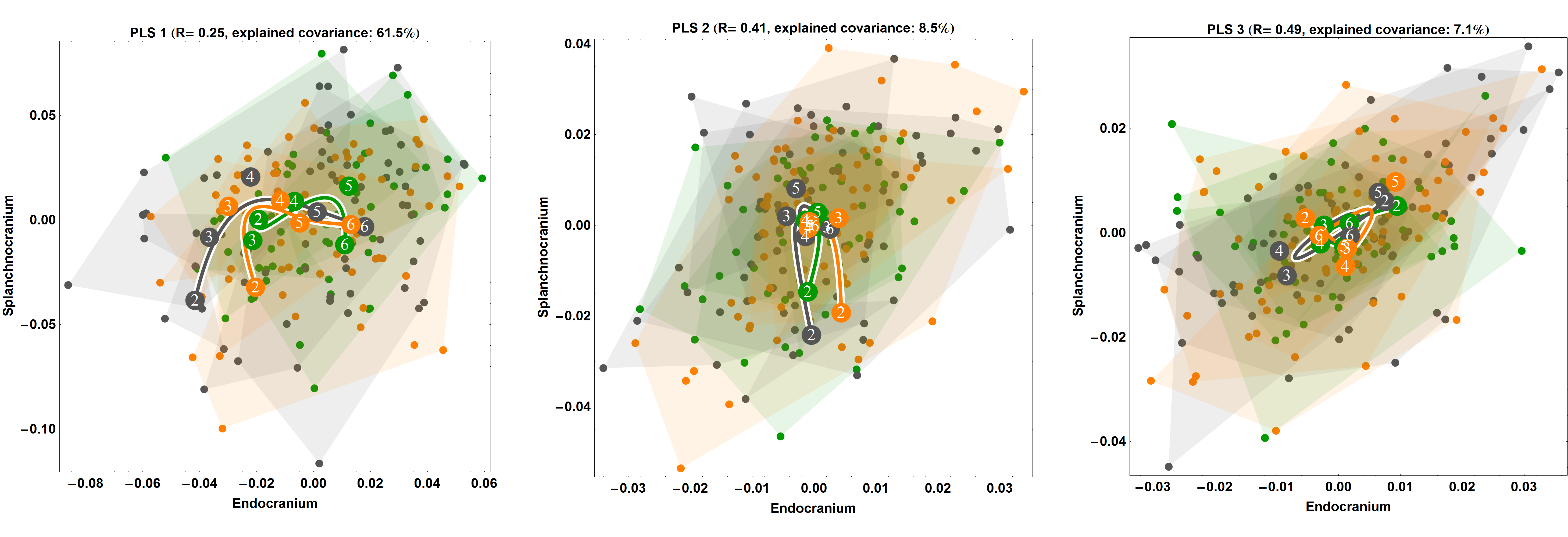

Supplement: S2 Fig — Convex hulls represent pooled sexes of age groups 2–6 for each species; age group labels denote age group means, while lines are B-spline curves of the average species-specific trajectories. Colours: green = chimpanzee; dark grey = gorilla; orange = orangutan. (TIFF) [file pone.0208999.s002.tiff]

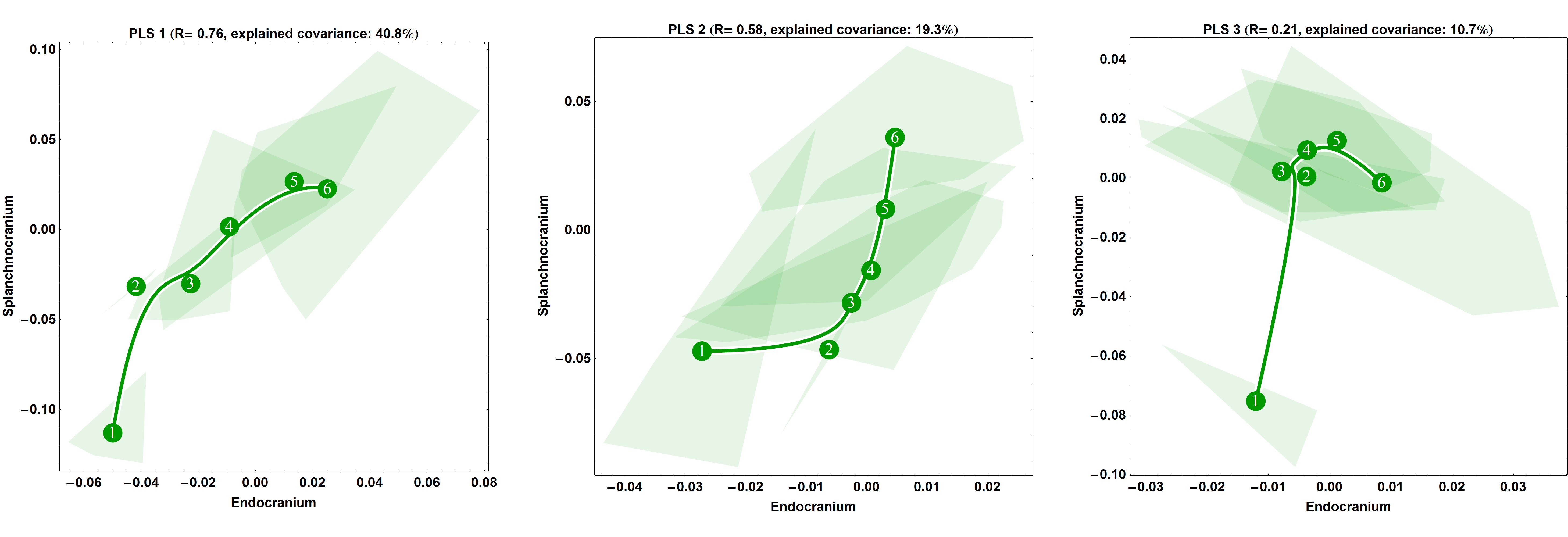

Supplement: S3 Fig — Convex hulls represent pooled sexes of age groups 1–6; age group labels denote age group means, while line is B-spline curve of the average chimpanzee-specific trajectory. (TIFF) [file pone.0208999.s003.tiff]

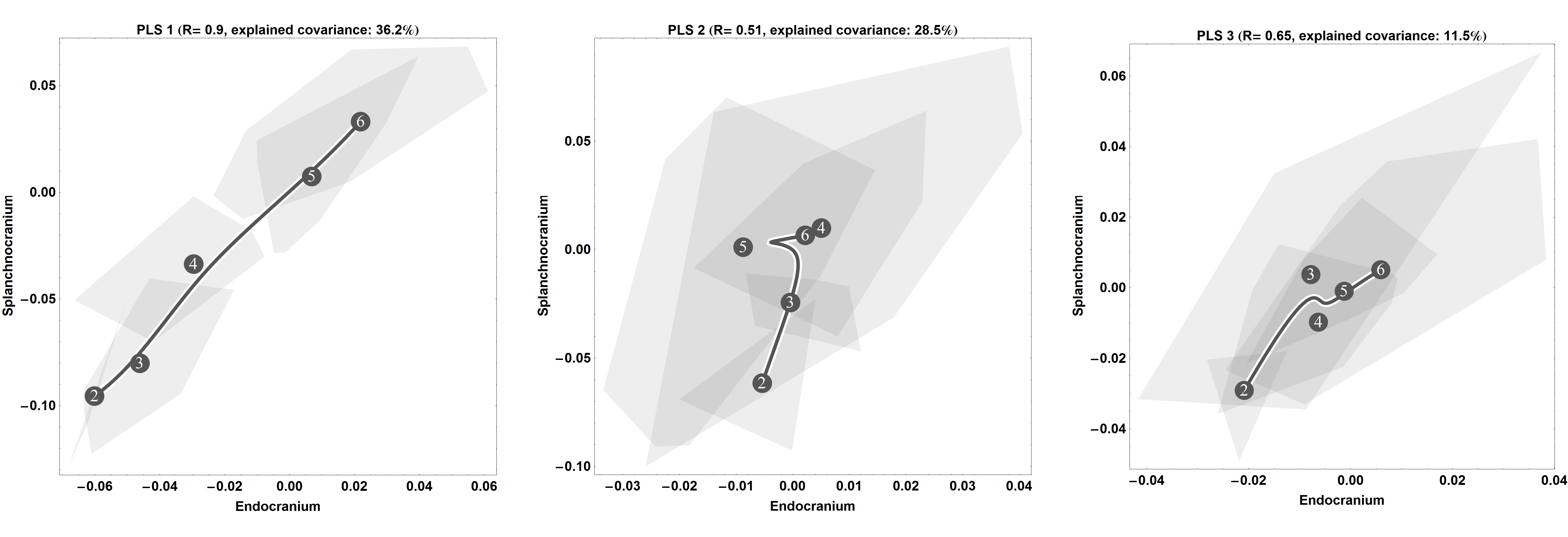

Supplement: S4 Fig — Convex hulls represent pooled sexes of age groups 2–6; age group labels denote age group means, while line is B-spline curve of the average gorilla-specific trajectory. (TIFF) [file pone.0208999.s004.tiff]

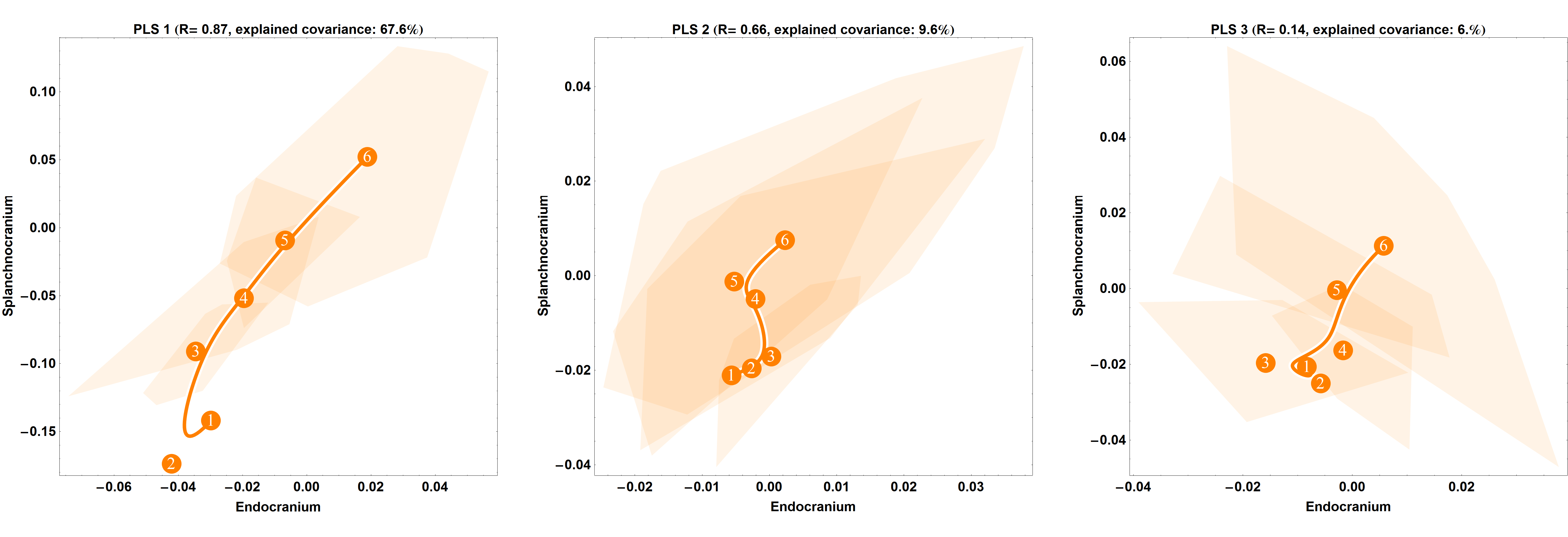

Supplement: S5 Fig — Convex hulls represent pooled sexes of age groups 1–6; age group labels denote age group means, while line is B-spline curve of the average orangutan-specific trajectory. (TIFF) [file pone.0208999.s005.tiff]
